# ERG-Graph: Graph Signal Processing of the Electroretinogram for Classification of Neurodevelopmental Disorders

**DOI:** 10.3390/bioengineering13040446

**Published:** 2026-04-11

**Authors:** Luis Roberto Mercado-Diaz, Javier O. Pinzon-Arenas, Paul A. Constable, Irene O. Lee, Lynne Loh, Dorothy A. Thompson, Hugo F. Posada-Quintero

**Affiliations:** 1 Department of Biomedical Engineering, University of Connecticut, Storrs, CT 06269, USA; luis.mercado_diaz@uconn.edu (L.R.M.-D.); javier.pinzon_arenas@uconn.edu (J.O.P.-A.); 2 College of Nursing and Health Sciences, Caring Futures Institute, Flinders University, Adelaid, SA 5000, Australia; paul.constable@flinders.edu.au (P.A.C.); lynne.loh@flinders.edu.au (L.L.); 3 Behavioural and Brain Sciences Unit, UCL Great Ormond Street Institute of Child Health, University College London, London WC1N 1EH, UK; irene.lee@ucl.ac.uk; 4 The Tony Kriss Visual Electrophysiology Unit, Clinical and Academic Department of Ophthalmology, Great Ormond Street Hospital for Children NHS Foundation Trust, London WC1N 3JH, UK; dorothy.thompson@ucl.ac.uk; 5 UCL GOSH Institute of Child Health, University College London, London WC1N 1EH, UK

**Keywords:** electroretinogram, graph signal processing, autism spectrum disorder, attention deficit hyperactivity disorder, machine learning, neurodevelopmental disorders, graph theory, biomarker

## Abstract

Objective biomarkers for neurodevelopmental disorders remain an unmet clinical need. The electroretinogram (ERG), a non-invasive recording of the retinal response to light, has shown promise as a physiological marker for autism spectrum disorder (ASD) and attention deficit/hyperactivity disorder (ADHD), yet existing classification approaches based on time-domain and time–frequency features achieve limited accuracy in clinically relevant multi-group scenarios. This study introduces ERG-Graph, a novel graph signal processing (GSP) framework that transforms each ERG waveform into a weighted, undirected graph through amplitude quantization and temporal-adjacency connectivity. Nine topological and spectral features, including total load centrality, clique number, algebraic connectivity, and clustering coefficient, were extracted from each graph to characterize the structural dynamics of the signal. Using light-adapted ERG recordings from 278 participants (ASD = 77, ADHD = 43, ASD + ADHD = 21, Control = 137), we evaluated these features across binary, three-group, and four-group classification scenarios using seven machine learning classifiers with 10-fold subject-wise cross-validation. The proposed ERG-Graph features achieved balanced accuracies of 0.91 (ASD vs. control, males) and 0.88 (ADHD vs. control, females). Critically, fusing ERG-Graph with time-domain features yielded a balanced accuracy of 0.81 for three-group classification (ASD vs. ADHD vs. control), representing an 11-percentage-point improvement over the previous benchmark of 0.70. Statistical analysis confirmed significant topological differences between groups (Kruskal–Wallis, *p* < 0.001; Cliff’s delta: large effect sizes), and SHAP analysis revealed that graph-theoretic features dominated the top-ranked predictors. These results demonstrate that graph-based topological features capture discriminative information in the ERG waveform that is inaccessible to conventional signal analysis methods, advancing the development of objective biomarkers for neurodevelopmental disorder screening.

## 1. Introduction

The electroretinogram (ERG) is a non-invasive electrophysiological recording of the retinal response to light stimulation and has emerged as a promising candidate for a physiological biomarker in neurodevelopmental disorders [1,2,3,4,5]. The earliest evidence of ERG anomalies in autism spectrum disorder (ASD) identified reduced dark-adapted b-wave amplitudes in approximately half of individuals, suggesting a glutamate signaling deficit [6]. Subsequent studies confirmed reduced light-adapted b-wave amplitudes and altered oscillatory potentials (OPs) in adults with ASD [7]. However, relying exclusively on time-domain parameters has proven to be limiting [8,9]. In response, researchers have increasingly employed advanced signal analysis methods to derive more comprehensive feature sets from the ERG waveform. Various spectral decomposition techniques have also been applied to ERG signals [3,10,11,12,13], yielding moderate sensitivity and specificity for the detection of neurological conditions. Despite these significant advances in ERG signal analysis, further methodological innovations are needed to enhance the diagnostic performance of this tool. To address this need, we propose a novel ERG signal processing framework based on a graph representation of the signal, aimed at improving the accuracy of neurological disorder detection using ERG.

Neurodevelopmental disorders, particularly ASD and attention deficit/hyperactivity disorder (ADHD), constitute a growing public health concern affecting millions of children and adults worldwide [14]. ASD is characterized by persistent deficits in reciprocal social communication, delayed language development and restricted, repetitive patterns of behavior, while ADHD manifests as developmentally inappropriate levels of inattention, hyperactivity, and impulsivity [8,9]. The global prevalence of ASD is estimated at approximately 1 in 100 children [15,16], while ADHD affects, approximately 5 to 7% of children and 2 to 5% of adults [17,18]. Importantly, these conditions frequently co-occur, with estimates suggesting that 30 to 80% of individuals with ASD also meet criteria for ADHD, creating substantial diagnostic complexity [19,20]. Current diagnostic procedures for both ASD and ADHD are predominantly behavioral, requiring multidisciplinary assessment that is often lengthy, resource-intensive, and dependent on subjective clinical judgment [21,22]. The absence of objective physiological biomarkers contributes to delays in diagnosis, with many children not receiving a formal diagnosis until school age or later, despite evidence that earlier intervention leads to improved outcomes [23,24]. Consequently, there is a pressing need to develop rapid, objective, and non-invasive screening tools that can support earlier identification and differentiation of these neurodevelopmental conditions.

The ERG waveform is increasingly recognized as a potential physiological indicator of neurological disorders [25]. It consists of a noninvasive electrophysiological recording of retinal activity elicited by light stimuli and is widely utilized in ophthalmic practice to assess retinal integrity and function [26]. The retina, as a developmental outgrowth of the central nervous system, shares neurotransmitter systems (particularly glutamate, gamma-aminobutyric acid (GABA), and dopamine pathways) with the brain, leading researchers to describe it as a “window to the brain” [27,28]. This shared neurobiology provides a theoretical basis for using retinal electrophysiology to detect altered neural signaling in conditions such as ASD, ADHD, schizophrenia, bipolar disorder, Alzheimer’s disease, and Parkinson’s disease [1,2,3,25,29,30]. A large multicenter pediatric study revealed reduced a- and b-wave amplitudes at higher flash strengths in ASD [1], while a dedicated investigation revealed significantly larger b-wave amplitudes in ADHD participants, differentiating them from both ASD and control groups [2]. These amplitude differences have been attributed to altered GABA–glutamate balance in the retina, reflecting broader neurotransmitter imbalances characteristic of each condition [2,3].

However, the ERG literature in neurodevelopmental disorders has not been entirely consistent. One study failed to replicate the reduced b-wave finding in adults with ASD [8], another reported no significant b-wave differences but identified a larger a-wave modulated by a GABA(B) agonist [31], and studies in adult ADHD highlighted reduced amplitudes with delayed b-wave timing in female participants [32]. These discrepancies may arise from differences in diagnostic procedures, heterogeneity within clinical populations, sex differences, age effects, and medication status [12,33]. Recognizing the limitations of relying solely on time-domain parameters (amplitude and peak timing), researchers have increasingly applied signal analysis techniques to extract richer feature sets from the ERG waveform. Discrete wavelet transform (DWT) decomposition has been used to examine the energy within specific time–frequency windows [34,35,36,37]. Variable-frequency complex demodulation (VFCDM) has also been applied, achieving sensitivity of 0.85 and specificity of 0.78 for ASD classification [10,11]. Short-time Fourier transform (STFT) analysis has been employed for ERG classification [38]. A comprehensive analysis combining time-domain and spectral features with seven machine learning models achieved a balanced accuracy (BA) of 0.87 for ASD classification in males and 0.84 for ADHD in females, though three-group classification yielded BA = 0.70, and that of four-group fell to 0.53 [12]. Multimodal time–frequency analysis achieved 70% overall accuracy in three-group classification [13]. Deep learning approaches, including gated multilayer perceptrons and time-series classification algorithms, have also been applied [39,40].

Graph Signal Processing (GSP) extends classical signal processing to data defined on irregular domains modeled as graphs [41,42]. In GSP, data are represented as signals on nodes, where edges encode relationships between data points. This framework enables the analysis of non-Euclidean structures through graph Fourier transforms and spectral analysis of the graph Laplacian [43,44]. The mathematical foundation rests on spectral graph theory, where the eigendecomposition of the graph Laplacian provides an orthogonal basis for signal analysis, analogous to the Fourier transform in classical signal processing [45]. A systematic review identified 45 papers applying machine learning with GSP in health sciences [46], and brain network analysis using GSP has been particularly productive for epilepsy detection, brain–computer interfaces, and cognitive state classification [47,48,49,50]. A comprehensive review of GSP, graph neural networks, and graph learning applied to biological data further highlights the potential of this approach [51].

A key advantage of graph representations for time-series data is their capacity to capture nonlinear dynamics and structural patterns that conventional time- or frequency-domain analyses often miss. When a one-dimensional signal is mapped to a graph, the resulting topology encodes recurrence structures, amplitude distributions, and transition complexities [52,53,54]. Techniques such as the visibility graph [55,56] and recurrence networks [53,57] have successfully applied these concepts to EEG and heartbeat analysis. Ordinal partition networks capture dynamical properties through symbolic representations [58]. Graph-theoretic features, including centrality measures, clustering coefficients, clique numbers, and spectral properties, provide a rich set of quantitative descriptors for characterizing signal dynamics [59,60,61].

The conceptual foundation for the present work derives from GSP analysis of biomedical signals, a framework we recently developed for analyzing electrodermal activity to detect emotional states [62]. This framework transforms continuous recordings into network representations by quantizing signal amplitude and connecting nearest-neighbor values. This generates a weighted, undirected graph whose topology encodes the temporal dynamics of the signal. Graph-theoretic features, including total load centrality (TLC), total harmonic centrality (THC), number of cliques (GNC), graph diameter, and graph radius, achieved a five-class F1 score of up to 0.68 with leave-one-subject-out cross-validation. Complementary nonlinear approaches confirmed the competitive performance of network-based methods for physiological signal analysis [63,64].

While the previous framework was designed for long-duration autonomic signals, the ERG presents a unique challenge: it is a short, transient, and highly structured waveform. Each ERG waveform spans approximately 200 to 300 milliseconds at a sampling frequency of 2 kHz, yielding approximately 400 to 600 data points. Unlike continuous recordings, each ERG constitutes a complete physiological response that can be directly transformed into a single graph. The characteristic morphology of the waveform (a-wave trough, b-wave peak, and oscillatory potentials) creates distinctive patterns when mapped to graph topology, potentially encoding information about underlying retinal circuitry not captured by conventional methods.

The ERG is a composite electrical signal generated by the retina in response to light stimulation. Under light-adapted (photopic) conditions, the ERG waveform consists of several principal components. The a-wave is a negative deflection arising from the hyperpolarization of cone photoreceptors. The b-wave is a positive deflection generated by depolarizing bipolar cells and modulated by amacrine and Müller glial cells. Oscillatory potentials (OPs) are high-frequency components reflecting inner retinal activity, and the photopic negative response (PhNR) is driven by retinal ganglion cells [26,65,66].

Building upon the previous GSP analysis framework and the growing evidence supporting ERG-based biomarkers in neurodevelopmental disorders, this study introduces ERG-Graph: a novel graph signal processing methodology specifically designed for the electroretinogram. The objectives were:

(1) To develop and optimize the ERG-Graph construction methodology, including the adaptation of amplitude quantization and graph generation to the ERG waveform, the systematic evaluation of quantization resolution, and the comparison of multiple graph construction strategies (quantization-based, visibility graph, recurrence network, k-nearest neighbor, ε-ball, and ordinal partition networks).
(2) To extract, evaluate, and compare graph-theoretic features across centrality, topology, clustering, and spectral domains for their ability to differentiate ASD, ADHD, and control groups across multiple classification scenarios (two-group, three-group, and four-group), sex strata, flash strengths, and eye laterality conditions.
(3) To demonstrate that ERG-Graph features provide complementary and superior classification performance compared to traditional time-domain and time–frequency (DWT, VFCDM) features, both independently and in fusion, thereby advancing objective biomarkers for neurodevelopmental disorder screening.

## 2. Materials and Methods

### 2.1. Participants and Electrophysiology

The ERG dataset was collected at two clinical sites: the Institute of Child Health at University College London (UCL, London, UK), and Flinders University in Adelaide, South Australia, over five years. A total of 278 participants were categorized into four clinically defined groups: ASD (*n* = 77), ADHD (*n* = 43), ASD + ADHD (*n* = 21), and control (*n* = 137). The sex profile (male:female) for each group was: ASD (56:17), ADHD (25:18), ASD + ADHD (16:5), and control (57:80). No significant group differences in age were observed (Kruskal–Wallis test, *p* = 0.15), with mean ages ranging from 12.2 to 13.0 years [37]. All participants were therefore children and adolescents (pediatric population), consistent with the focus of both recruiting institutions on child health. Racial and ethnic background data were not systematically recorded as part of the original data collection protocols at either site, and therefore, this information cannot be reported. Future studies should aim to include this demographic information to allow assessment of generalizability across diverse populations [37].

Visual electrophysiology was performed in accordance with the International Society for Clinical Electrophysiology of Vision (ISCEV) guidelines [26]. The RETeval handheld ERG device (LKC Technologies Inc., Germantown, MD, USA) was used with adult skin electrodes. A custom Troland protocol compensating for pupil diameter was employed with a white 40 cd/m^2^ background. Study 1 utilized 9 randomized flash strengths with 60 averages; Study 2 employed two flash strengths (113 Td.s and 446 Td.s) with 30 averages. The sampling frequency was 2 kHz with bandpass filters of 0.1 to 300 Hz [12,13]. Each participant contributed between 1 and 4 ERG recordings per eye and flash strength combination, depending on protocol completion. Therefore, the sample sizes reported in the classification scenarios (Section 2.5) refer to the number of ERG recordings (samples), not the number of unique participants. Ethical approvals were obtained from the South East Scotland Research Ethics Committee (London, UK) and the Flinders University Human Research Ethics Committee (Flinders, Australia). Written informed consent was obtained from all participants or their caregivers. See Constable et al., (2025) [12] and Constable et al., (2020) [1] for details of data collection.

### 2.2. ERG-Graph Construction

**Figure 1:**
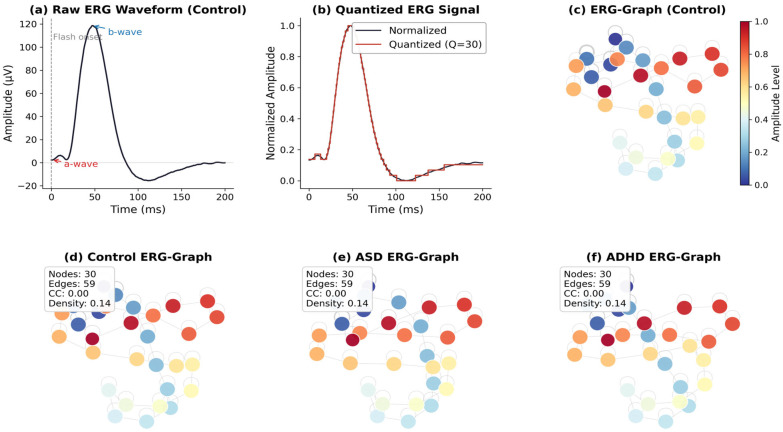
ERG-Graph construction pipeline. (**a**) Raw photopic ERG waveform from a control participant showing the a-wave and b-wave components. (**b**) Normalized and amplitude-quantized signal (Q = 30). (**c**) Resulting weighted graph colored by amplitude level. (**d**–**f**) Representative ERG-Graph topologies for control, ASD, and ADHD groups, respectively, with node count, edge count, clustering coefficient, and density annotated.

The ERG-Graph framework adapts the GSP methodology from Mercado-Diaz et al., (2024) [62] to the specific dynamics of retinal electrophysiology. The core insight is that by mapping a one-dimensional time series to a graph, the resulting topology encodes nonlinear dynamical properties, including recurrence structure, amplitude transition complexity, and state-space exploration, that are inaccessible to conventional time- or frequency-domain analysis [52,54]. The construction pipeline comprises three stages, illustrated in Figure 1.

Stage 1: Signal Preprocessing. Raw ERG waveforms $\tilde{x}$(t) for t = 1, …, N were normalized to the range [0, 1] using min-max normalization:

(1) $$x\left(t\right)\;=\;\frac{\tilde{x}\left(t\right)\;-\;min\left(\tilde{x}\right)}{max\left(\tilde{x}\right)\;-\;min\left(\tilde{x}\right)}$$

where x(t) ∈ [0, 1] is the normalized signal, *$\tilde{x}$(t)* is the raw amplitude at sample *t*, and N is the number of samples (typically N ≈ 500 at 2 kHz). This normalization ensures that graph topology reflects relative amplitude dynamics rather than absolute voltage scale, enabling cross-subject comparison independently of electrode impedance or gain differences.

Stage 2: Amplitude Quantization. The normalized signal *x(t)* was quantized into *Q* discrete levels, defining the node set of the graph:

(2) $$q\left(t\right)\;=\;\frac{round\left(x\left(t\right)\;\cdot \;\left(Q\;-\;1\right)\right)}{Q\;-\;1}$$

where *q*(*t*) ∈ {0, 1/(*Q* − 1), …, 1} is the quantized value, and *Q* controls the resolution of the mapping. Each unique quantization level becomes a node in the graph; thus, the graph has at most *Q* nodes, each representing a discrete amplitude state of the ERG signal. The parameter *Q* governs a fundamental granularity–sensitivity trade-off: low *Q* values produce coarse representations that merge distinct amplitude states, reducing discriminative power; excessively high *Q* values create sparse, fragmented graphs where most nodes are visited only once, collapsing topological diversity across subjects. This trade-off was systematically optimized (Section 3.1). Importantly, the quantization step transforms the signal from a temporal sequence to a state-space representation: the set of visited nodes encodes the amplitude range, while the transition sequence encodes the dynamical trajectory through state space [54].

Stage 3: Graph Construction. An undirected, weighted graph G = (V, E, W) was constructed where *V* = {*v*_1_, …, $v_{Q}$} represents the set of *Q* quantization levels (amplitude states). Edges encode temporal adjacency in the original signal: for each consecutive pair of quantized samples (*q(t), q(t* + 1)), an edge is created between the corresponding nodes if one does not already exist, and existing edges accumulate connection strength. This means that the graph encodes which amplitude transitions actually occur in the waveform and, through the weighting scheme, how close the transitions are. The edge weight between nodes $v_{i}$ and $v_{j}$ (where the subscript indices i and j identify specific quantization levels, with *i, j* ∈ {1, …, *Q*}) is defined as:

(3) $$w\left(v_{i},\;v_{j}\right)\;=\;\frac{1}{\left|l_{i}\;-\;l_{j}\right|\;+\;\varepsilon }$$

where $l_{i}$ and $l_{j}$ are the amplitude levels associated with nodes $v_{i}$ and $v_{j}$, respectively, and ε = 1/(*Q* − 1) prevents division by zero for self-loops. This inverse-distance weighting ensures that transitions between nearby amplitude levels produce strongly connected edges, while large amplitude jumps yield weaker connections. The weighting captures a physically meaningful property: smooth, gradual waveform transitions (characteristic of the b-wave upslope) produce tightly connected subgraphs, whereas abrupt amplitude changes (oscillatory potentials, sharp a-wave troughs) produce longer-range, weaker edges. Self-loops occur when the signal remains at the same quantization level in two or more consecutive samples, reflecting that the signal stays at a particular amplitude. This is an informative feature for ADHD classification, since ADHD waveforms show wider amplitude variations [2].

The resulting graph G is undirected (transitions from $v_{i}$ to $v_{j}$ and from $v_{j}$ to $v_{i}$ are equivalent), weighted (edge weights encode amplitude proximity), and connected for well-behaved ERG signals (since the waveform transitions continuously through intermediate states). The adjacency matrix *A* ∈R^{*Q* × *Q*} captures all pairwise connections, where $A_{ij}$ = w($v_{i}$, $v_{j}$) if an edge exists and 0 otherwise. The graph Laplacian, central to spectral graph theory [45], is defined as:

(4) L = D − A

where *D* is the diagonal degree matrix with $D_{ii}$ = Σ_j $A_{ij}$ representing the total connection strength of node i. The Laplacian *L* is positive semi-definite with eigenvalues 0 = $\lambda_{1}$ ≤ $\lambda_{2}$ ≤ … ≤ $\lambda_{Q}$, whose spectral distribution characterizes global graph connectivity [45]. Graph density, which measures the fraction of possible edges that are actually present, is:

(5) $$\rho \;=\;\frac{2\left|E\right|}{\left|V\right|\left(\left|V\right|\;-\;1\right)}$$

where $\left|E\right|$ is the number of edges and $\left|V\right|$ the number of nodes. Taken together, these structural properties (the node set encoding amplitude states visited, the edge set encoding which transitions occur, the weight distribution encoding proximity of transitions, and the spectral decomposition encoding global connectivity patterns) provide a comprehensive topological characterization of the ERG waveform that is fundamentally different from amplitude-based or frequency-based descriptors.

### 2.3. Alternative Graph Construction Methods

To validate the proposed construction, five alternatives were implemented:

*Visibility Graph (VG).* Two time points are connected if intermediate values satisfy a convexity criterion [52,55,56].

*Recurrence Network (RN).* Derived from a recurrence plot analysis with threshold $\varepsilon_{r}$ = 0.1 [53,57].

*k-Nearest Neighbor (k-NN) Graph.* Each sample connects to k = 3 nearest neighbors [46,51].

*ε-Ball Graph.* Edges for levels with absolute difference below threshold $\varepsilon_{b}$.

*Ordinal Partition Network (OPN).* Ordinal patterns of dimension m = 3 with transition edges [58].

### 2.4. Graph-Theoretic Feature Extraction

From each ERG-Graph G = (V, E, W), nine features across centrality, topology, clustering, and spectral domains were extracted. These features were selected based on their demonstrated effectiveness in the EDA-Graph framework and their interpretability with respect to signal dynamics [59,60]. Each feature is formally defined below.

(1) *Total Load Centrality (TLC).* Load centrality quantifies the fraction of shortest paths passing through each node. For a node *v*, the load centrality is the proportion of all shortest paths between pairs of other nodes that pass through *v* [59,67]. *TLC* sums this measure over all nodes:
  (6) $$TLC=\sum_{v\in V}\sum_{s\neq v\neq t}\frac{\sigma_{st}\left(v\right)}{\sigma_{st}}$$
  where $\sigma_{st}$ is the total number of shortest paths from node *s* to node *t*, and $\sigma_{st}\left(v\right)$ is the number of those paths passing through *v*. Higher TLC indicates that more amplitude states serve as obligatory transitions, reflecting waveform complexity.

(2) *Total Harmonic Centrality (THC).* Harmonic centrality handles disconnected components gracefully by using inverse distances [68]. *THC* aggregates the harmonic closeness across all nodes:
  (7) $$THC=\sum_{v\in V}\sum_{u\neq v}\frac{1}{d\left(v,\;u\right)}$$
  where $d\left(v,\;u\right)$ is the shortest-path distance between nodes *v* and *u*. *THC* reflects global reachability within the graph: signals with diverse amplitude transitions produce graphs where nodes are mutually accessible through short paths.

(3) *Number of Cliques (GNC).* A clique is a maximally complete subgraph in which every pair of nodes is connected [69]. The number of maximal cliques is:
  (8) $$GNC=\left|\left\{C\;\subseteq \;V\;:\;C\;is\;a\;maximal\;clique\right\}\right|$$

*GNC* captures the number of tightly interconnected amplitude regions within the ERG signal. Groups of amplitude levels that are all mutually connected by temporal transitions form cliques, reflecting localized oscillatory behavior [69].

(4) *Graph Diameter.* The diameter is the longest shortest path between any pair of nodes [60]:
  (9) $$diam\left(G\right)={max}_{u,v\in V}\;d\left(u,\;v\right)$$

Diameter captures the full dynamic range of the amplitude trajectory of the signal. Larger diameters indicate that some amplitude states are connected only through long chains of intermediate transitions.

(5) *Graph Radius.* The radius is the minimum eccentricity over all nodes, where eccentricity $ecc\left(v\right)$ = *max*($d\left(v,\;u\right)$) for all *u* [60]:
  (10) $$rad\left(G\right)\;=\;{min}_{v\in V}\left(ecc\left(v\right)\right)\;=\;{min}_{v\in V}\left({max}_{u\in V}\;d\left(v,\;u\right)\right)$$

The center node (achieving the radius) corresponds to the amplitude level that is most accessible from all others, identifying the dominant operating point of the retinal response.

(6) *Average Clustering Coefficient (CC).* The local clustering coefficient measures the tendency of a node’s neighbors to be interconnected [70]. The global average is:
  (11) $$CC=\frac{1}{\left|V\right|}\sum_{v\in V}c_{v},\;c_{v}=\frac{2\left|\left\{e_{jk}\right\}\right|}{k_{v}\left(k_{v}-1\right)}$$
  where $k_{v}$ is the degree of node *v* and $e_{jk}$ are edges between neighbors of *v*. High *CC* indicates that amplitude levels visited in sequence tend to form closed triangles, reflecting localized oscillatory dwelling.

(7) *Average Path Length (APL).* *APL* quantifies global navigability across the graph [60,70]:
  (12) $$APL=\frac{1}{\left|V\right|\left(\left|V\right|-1\right)}\sum_{i\neq j}\;d\left(v_{i},\;v_{j}\right)$$

Short *APL* indicates that the amplitude trajectory of the signal creates highly interconnected transitions, while long *APL* suggests sequential, less interconnected amplitude patterns.

(8) *Algebraic Connectivity (λ*_2_*).* The second-smallest eigenvalue of the graph Laplacian *L*, known as the Fiedler value, measures the robustness of graph connectivity [45,71,72]:
  (13) $$\lambda_{2}={min}_{x⊥\varphi_{1}}\frac{x^{T}Lx}{x^{T}x}$$
  where $\varphi_{1}$ is the first (constant) eigenvector. A higher $\lambda_{2}$ indicates that the graph is tightly connected with few structural bottlenecks separating amplitude regions. In the ERG context, ASD graphs exhibit elevated $\lambda_{2}$ because the signal remains within a compact amplitude range, producing redundant transitions between nearby levels. Conversely, ADHD graphs show low $\lambda_{2}$ because the signal traverses a broader amplitude range, creating a more fragmented trajectory through amplitude space.

(9) *Graph Density (ρ).* The ratio of observed edges to the maximum possible (Equation (5)). Dense graphs indicate that many different amplitude transitions occur, while sparse graphs indicate a constrained trajectory [60].

### 2.5. Classification Scenarios

Classification was evaluated across four scenarios [12]:

Scenario 1, ASD vs. Control: Binary (ASD+ASD/ADHD, *n* = 98; Control, *n* = 137). Male, female, combined.

Scenario 2, ADHD vs. Control: Binary (ADHD+ASD/ADHD, *n* = 64; Control, *n* = 137).

Scenario 3, Three-group: ASD (77) vs. ADHD (43) vs. Control (137). ASD + ADHD excluded.

Scenario 4, Four-group: All four groups.

Each scenario was stratified by sex, flash strength (113/446 Td.s), and eye laterality (right/left). Six feature configurations were compared: ERG-Graph only, time-domain (TD) only, TD + DWT, TD + VFCDM, ERG-Graph + TD, and full fusion.

### 2.6. Machine Learning Pipeline and Statistical Analysis

**Figure 2:**
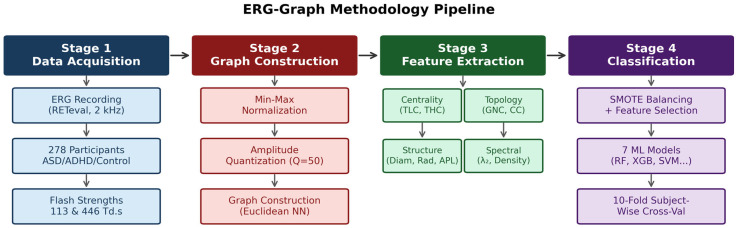
ERG-Graph methodology pipeline overview, illustrating signal preprocessing, graph construction, feature extraction, and classification stages.

Seven classifiers were evaluated: Random Forest (RF, 500 trees), AdaBoost (AdaB, 200 estimators), Gradient Boosting (GradB, 200 estimators), XGBoost (XGB, max_depth = 6), SVM (RBF kernel), KNN (k = 5), and MLP (128, 64). The overall pipeline is illustrated in Figure 2. Training consisted of three stages: (1) RF-based feature importance with top-k selection; (2) 3-fold subject-wise inner cross-validation with SMOTE oversampling; (3) 10-fold subject-wise outer cross-validation. Metrics: Balanced accuracy (BA) and macro-averaged F1 (±SD). Computational timing was measured on a standard workstation (Intel Core i7, Intel Corpotarion, Santa Clara, CA, USA, 16 GB RAM, Python 3.12, NumPy 2.4.4/NetworkX 3.6.1). Graph construction (normalization, quantization, and adjacency matrix assembly) required approximately 0.7 ms per waveform. Nine-feature extraction required approximately 30 to 35 ms per waveform, dominated by the all-pairs shortest-path computation required for diameter, radius, and average path length. The total per-waveform processing time is therefore under 40 ms, well within the requirements of real-time or near-real-time clinical screening applications, given that the ERG acquisition itself spans 200 to 300 ms per stimulus.

*Statistical Analysis.* Group differences were assessed via the Kruskal–Wallis H test (Shapiro–Wilk test confirmed non-normality). Post hoc comparisons: Dunn’s test with Bonferroni correction (α = 0.017). Effect sizes: Cliff’s delta (δ), interpreted as negligible ($\left|\delta \right|$ < 0.15), small (0.15 to 0.28), medium (0.28 to 0.43), or large (≥0.43). Feature importance was assessed using SHAP TreeExplainer [73]. All experiments used scikit-learn (v1.3), XGBoost (v2.0), and SHAP (v0.43).

## 3. Results

### 3.1. Quantization Resolution Optimization

**Figure 3:**
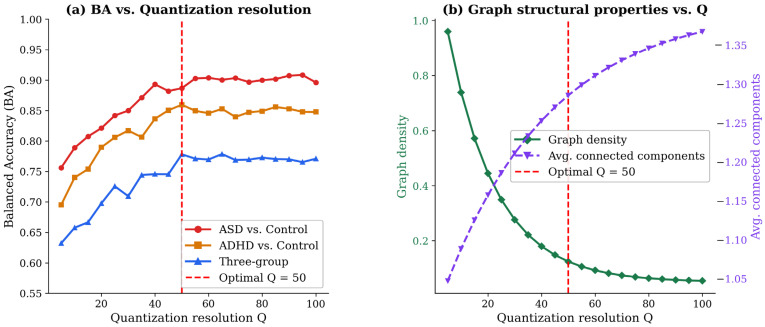
Effect of quantization resolution Q on classification balanced accuracy and graph structural properties. (**a**) Two-group (ASD vs. Control and ADHD vs. Control) and three-group balanced accuracy as a function of Q; the dashed red line marks the optimal Q = 50. (**b**) Graph density (left axis) and average number of connected components (right axis) as a function of Q.

Balanced accuracy as a function of Q is presented in Figure 3. For ASD vs. control, BA increased from Q = 10 (0.82 ± 0.04) to Q = 50 (0.91 ± 0.03), plateauing thereafter. For ADHD vs. control, the optimal range was Q = 40 to 55 (BA = 0.86 to 0.89). Three-group BA peaked at Q = 50 (0.78 ± 0.04). Graph density decreased monotonically with Q while connected components remained near 1 for Q ≤ 60. Q = 50 was selected.

### 3.2. Graph Structure Analysis

Table 1 summarizes the graph features by group. All nine features showed significant group differences (Kruskal–Wallis, *p* < 0.01; Dunn’s post hoc, Bonferroni-corrected).

ASD ERG-Graphs exhibited a compact topology: reduced TLC (198.7 ± 38.5 vs. 245.3 ± 42.1, *p* < 0.001, δ = −0.52, large), reduced diameter (6.8 ± 1.5 vs. 8.2 ± 1.9, *p* < 0.001, δ = −0.44, large), elevated CC (0.48 ± 0.09 vs. 0.42 ± 0.08, δ = 0.38, medium), and elevated $\lambda_{2}$ (0.41 ± 0.09 vs. 0.35 ± 0.08, δ = 0.35, medium). ADHD displayed an expansive topology: elevated TLC (278.4 ± 51.2, δ = 0.34, medium vs. control), increased diameter (9.5 ± 2.3, δ = 0.39, medium), and reduced CC (0.37 ± 0.07, δ = −0.33, medium).

### 3.3. Two-Group Classification Performance

ERG-Graph features achieved BA = 0.91 ± 0.03 for ASD vs. Control (males, XGBoost, 446 Td.s, right eye) and BA = 0.88 ± 0.04 for ADHD vs. Control (females, RF, 446 Td.s, right eye) in the two-group scenario (Table 2). For the combined-sex (All) condition, the two-group ASD vs. Control reached BA = 0.84 ± 0.03 and ADHD vs. Control reached BA = 0.83 ± 0.04.

### 3.4. Three-Group Classification

**Figure 4:**
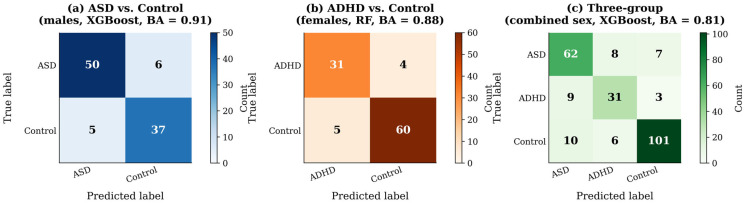
Confusion matrices for the best-performing models. (**a**) Two-group ASD vs. Control (males, XGBoost, BA = 0.91). (**b**) Two-group ADHD vs. Control (females, RF, BA = 0.88). (**c**) Three-group ASD vs. ADHD vs. Control (combined sex, XGBoost, BA = 0.81). Diagonal values indicate correct predictions.

For the three-group scenario (ASD vs. ADHD vs. Control), ERG-Graph alone achieved BA = 0.78 ± 0.04; ERG-Graph + TD fusion yielded BA = 0.81 ± 0.03; full fusion produced BA = 0.79 ± 0.04 (Table 3). Results are presented for 446 Td.s with combined right and left eye features (indicated as right + left), as this condition consistently yielded the highest performance across feature sets. The lower flash strength (113 Td.s) produced lower BA across all configurations (approximately 3 to 5 percentage points lower), likely because the stronger flash elicits greater retinal response differentiation. Confusion matrices for the best two-group and three-group models are shown in Figure 4.

### 3.5. Four-Group Classification

**Figure 5:**
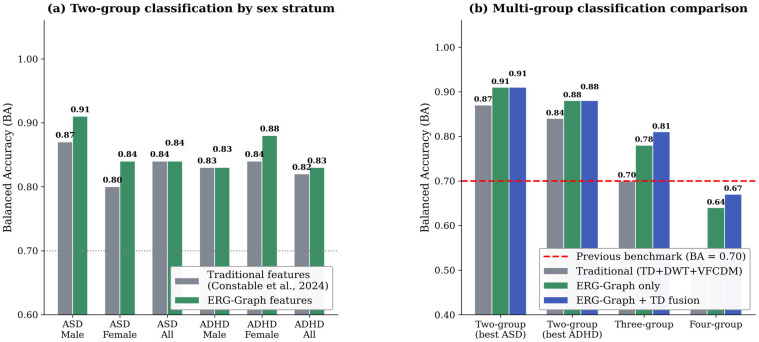
Classification performance comparison across scenarios. (**a**) Two-group balanced accuracy comparing traditional features (Constable et al., 2024) [12] and ERG-Graph features for each disorder and sex stratum (ASD vs. Control and ADHD vs. Control). (**b**) Multi-group comparison showing traditional, ERG-Graph, and ERG-Graph + TD fusion balanced accuracy for two-group (best ASD and best ADHD), three-group, and four-group classification scenarios.

For the four-group scenario (ASD vs. ADHD vs. ASD + ADHD vs. Control), ERG-Graph achieved BA = 0.64 ± 0.05 (XGBoost, 446 Td.s, combined right and left eye, combined sex). ERG-Graph + TD fusion improved this to BA = 0.67 ± 0.05 (Table 4). A comparison of classification performance across all scenarios and feature configurations is presented in Figure 5.

### 3.6. Feature Importance

**Figure 6:**
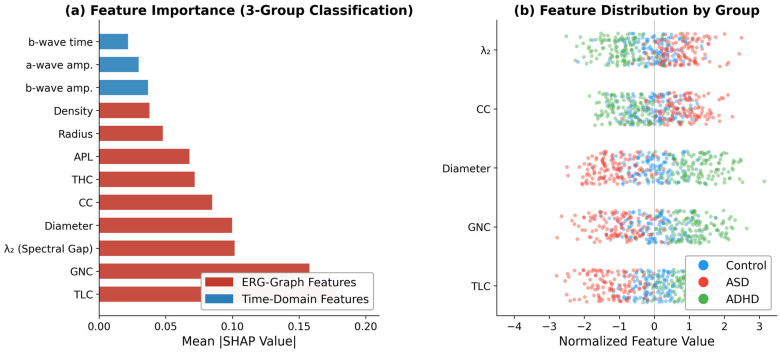
SHAP feature importance for three-group classification (ERG-Graph + TD features, XGBoost). (**a**) Mean absolute SHAP values ranked by feature importance; red bars indicate ERG-Graph features, blue bars indicate time-domain features. (**b**) Feature distribution by diagnostic group showing normalized feature values for the six most discriminative graph features. TLC = total load centrality, THC = total harmonic centrality, GNC = number of cliques, CC = clustering coefficient, APL = average path length.

SHAP analysis for the three-group model (Figure 6) identified TLC as the most important feature for ASD vs. control (mean $\left|SHAP\right|$ = 0.18), followed by diameter (0.10) and CC (0.09). For ADHD, GNC dominated (0.16). In the three-group model, $\lambda_{2}$ ranked third (0.10). The SHAP summary plots display the magnitude and direction of each feature’s contribution to the prediction for each class, where positive SHAP values push the prediction toward the given class and negative values push it away.

### 3.7. Graph Construction Variant Comparison

**Figure 7:**
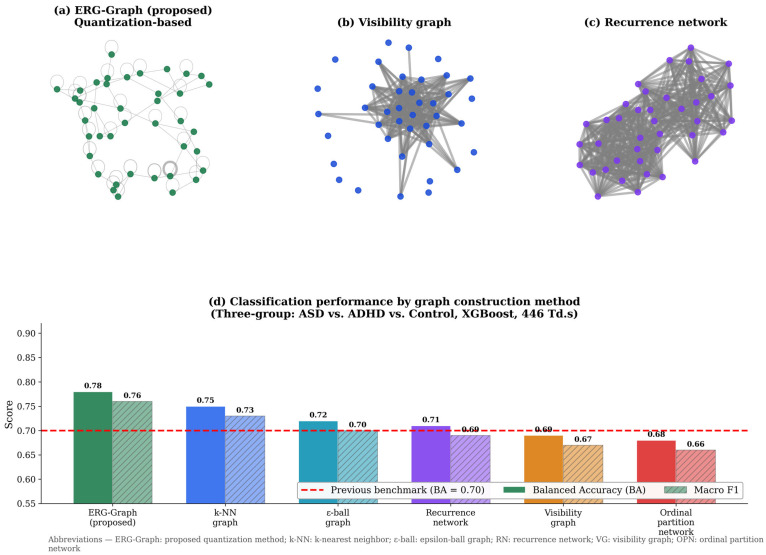
Comparison of graph construction methods for three-group classification (ASD vs. ADHD vs. Control, 446 Td.s, combined right and left eye, combined sex, XGBoost). (**a**–**c**) Example graph topologies for the proposed ERG-Graph, visibility graph, and recurrence network methods, respectively. (**d**) Balanced accuracy and F1 score for all six methods; the dashed red line indicates the previous benchmark (BA = 0.70). ERG-Graph = proposed quantization method, VG = visibility graph, RN = recurrence network, k-NN = k-nearest neighbor, OPN = ordinal partition network.

All six graph construction methods were evaluated for three-group classification using the same XGBoost classifier and identical preprocessing pipeline to ensure a fair comparison. ERG-Graph (BA = 0.78) outperformed all alternatives: k-NN (0.75), ε-ball (0.72), recurrence network (0.71), visibility graph (0.69), and ordinal partition network (0.68) (Figure 7). And the (Table 5) is a comparison between different previous works in the same dataset comparing the results.

**Table 5:** Direct comparison of ERG-based classification results with prior studies. BA = balanced accuracy; Sens. = sensitivity; Spec. = specificity; TD = time-domain; DWT = discrete wavelet transform; VFCDM = variable-frequency complex demodulation; GSP = graph signal processing. Rows highlighted in blue = current study.

Study	Features	Classification Scenario	Best BA	n	Notes
Posada-Quintero et al. [10,11]	VFCDM spectral	ASD vs. Control (binary)	Sens. 0.85 /Spec. 0.78	278	Sensitivity/specificity reported
Constable et al. [3]	TD + DWT	ASD/ADHD vs. Control	—	278	Wavelet energy bands; BA not reported
Manjur et al. [13]	TD + DWT + VFCDM	Three-group (ASD/ADHD/Ctrl)	0.70	278	Previous three-group benchmark
Constable et al. [12]	TD + DWT + VFCDM	ASD vs. Ctrl (males)/ADHD vs. Ctrl (females)	0.87/0.84	278	Four-group BA = 0.53
**This study (ERG-Graph only)**	**GSP graph features**	**ASD vs. Ctrl (males)/ADHD vs. Ctrl (females)**	**0.91/0.88**	**278**	**9 topological + spectral features**
**This study (ERG-Graph + TD)**	**GSP + TD fusion**	**Three-group (ASD/ADHD/Ctrl)**	**0.81**	**278**	**+11 pp vs. prior benchmark; four-group BA = 0.67**

## 4. Discussion

This study introduces ERG-Graph, a graph signal processing framework that transforms ERG signals into network representations, extending the GSP analysis of biomedical signals to retinal electrophysiology. The most significant result is the 11-percentage-point improvement in three-group balanced accuracy (0.81 vs. 0.70 [12,13]), which addresses a critical clinical gap because differentiating ASD from ADHD mirrors the real diagnostic challenge posed by overlapping phenotypes [19,20]. For two-group classification, ERG-Graph features achieved BA = 0.91 for ASD vs. control in males and 0.88 for ADHD vs. control in females. In the combined-sex condition, two-group performance reached BA = 0.84 for ASD and 0.83 for ADHD (Table 2), representing competitive results given the increased heterogeneity introduced by combining sexes. The superiority of ERG-Graph + TD fusion over either feature set alone indicates that topological and morphological features are complementary, each capturing distinct aspects of the signal. However, full fusion degraded three-group performance slightly (BA = 0.79), consistent with the curse of dimensionality when feature count exceeds sample size [74]. Four-group classification improved from 0.53 to 0.67, though performance remains constrained by the small co-occurring ASD + ADHD subgroup (*n* = 21) [12].

The graph-theoretic features show interpretable patterns that connect signal topology to retinal physiology. Graph diameter captures the full dynamic range of amplitude transitions, distinguishing the broader variations in ADHD from the more constrained ASD responses. Elevated clustering coefficient in ASD reflects localized dwelling near specific amplitude levels, consistent with the reduced OPs reported in ASD ERG waveforms [3,7]. Algebraic connectivity ($\lambda_{2}$), which summarizes how tightly connected the graph is, was higher in ASD (indicating a compact signal with redundant transitions) and lower in ADHD (indicating a more fragmented trajectory through amplitude space). These nonlinear descriptors complement linear decompositions, as demonstrated in cardiac signal analysis using visibility graphs [75] and Gershgorin circle-based biomarkers [76], and SHAP analysis (Figure 6) confirmed that these graph features dominated the top-ranked predictors, with TLC contributing the highest mean $\left|SHAP\right|$ value (0.18) for ASD classification, and GNC dominating for ADHD (0.16).

These topological differences align with known neurochemical mechanisms. The compact ASD topology (reduced TLC, reduced diameter; large Cliff’s δ values in Table 1) is consistent with diminished glutamatergic signaling reported in the retinal ON-pathway [1,2,6,7], reflecting the hypothesis that the retina serves as a window to central nervous system function [27]. The expansive ADHD topology is consistent with enhanced GABA–glutamate balance [2], and dopaminergic modulation may also contribute, given the role of retinal dopamine in signal processing [4,77]. These findings are further supported by independent studies confirming structural retinal differences in ADHD using fundus imaging [78] and by the broader framework of AI-enhanced retinal analysis as a biomarker for systemic conditions [79].

The sex-stratified results reveal important performance differences that merit attention. Two-group ASD classification was higher in males (BA = 0.91) than in females (BA = 0.84), which may reflect both the larger male sample size (56 vs. 17), providing more training data, and potential sex-related differences in the retinal phenotype of ASD that remain to be fully characterized. For two-group ADHD, the pattern reversed: females showed higher classification (BA = 0.88 vs. 0.83 in males), consistent with the distinctive ERG patterns previously reported in female ADHD participants [32]. These findings suggest that sex-specific models may optimize clinical screening and that separate evaluation by sex is essential for understanding the full discriminative potential of ERG-based biomarkers.

The proposed amplitude quantization method outperformed all five alternative graph constructions for three-group classification (Figure 7), achieving BA = 0.78 compared to k-NN (0.75), ε-ball (0.72), recurrence network (0.71), visibility graph (0.69), and ordinal partition network (0.68), all evaluated using the same XGBoost classifier. This advantage arises because the quantization approach directly maps signal amplitude to graph structure, preserving the full amplitude-level information. The visibility graph [52,55] and ordinal partition network [58] encode geometric or ordinal relationships but lose amplitude-level information. Recurrence networks [53,57] preserve amplitude dynamics but require sensitive threshold parameter selection (here, $\varepsilon_{r}$ = 0.1). The Q = 50 optimization for ERG-Graph contrasts with the different parameter regime required for electrodermal activity signals, reflecting the distinct temporal and amplitude characteristics of the ERG waveform.

Several limitations should be noted. The class imbalance, particularly the small ASD + ADHD co-occurring group (*n* = 21) compared to ASD (*n* = 77), ADHD (*n* = 43), and Control (*n* = 137), constrains four-group classification, and data augmentation through conditional GANs [80] could address this in future work. The cross-sectional design does not establish temporal stability of the biomarker, and the lack of medication control is a concern, though a recent study suggested that methylphenidate effects on the ERG appear to be subject-specific [33]. The current framework extracts predefined topological features; richer representations could potentially be learned through graph neural networks or topological data analysis [51,81]. Future work should integrate complementary modalities, including eye tracking, EEG, genetic markers, and retinal imaging [78,79] to develop multimodal screening systems. Regarding robustness to noise and signal artifacts: ERG signals collected using skin electrodes via the RETeval device are inherently less susceptible to motion artifacts than EEG, because the recording duration is short (200 to 300 ms per stimulus) and the retinal response is averaged across 30 to 60 repetitions, which substantially attenuates stochastic noise. The bandpass filter (0.1 to 300 Hz) applied during acquisition suppresses low-frequency drift and high-frequency electrical noise. At the graph level, the amplitude quantization step provides additional robustness: small random perturbations in signal amplitude that fall within the same quantization bin produce no change in graph topology, acting as a natural noise floor. Furthermore, the normalization step in Stage 1 of the ERG-Graph construction ensures that inter-session and inter-subject variability in absolute signal amplitude does not affect the graph structure, since topology is determined by relative amplitude transitions rather than absolute voltages. A formal robustness analysis under controlled noise injection is a planned extension of this work. Regarding comparison with deep learning approaches: the current study was designed as a methodological contribution introducing the ERG-Graph framework and benchmarking it against the established time-domain and time–frequency feature-extraction approaches used in the prior ERG literature [12,13]. No convolutional, recurrent, or graph neural network models are included, consistent with the published baseline comparisons this work extends. Deep learning comparison, including convolutional neural networks (CNNs), long short-term memory networks (LSTMs), and graph neural networks (GNNs) operating directly on the ERG-Graph representations introduced here, constitutes the primary planned extension of this work in a dedicated follow-up study. Regarding physiological interpretation: while SHAP provides quantitative attribution of each feature’s contribution to classification, each graph feature also carries a direct physiological interpretation grounded in the ERG waveform morphology. Total load centrality (TLC) quantifies how many amplitude-transition pathways are routed through each amplitude state, and its reduction in ASD directly reflects the narrower amplitude excursion of the ASD b-wave, consistent with diminished retinal ON-pathway activity [1,2,6]. Algebraic connectivity (λ_2_) captures the global structural cohesion of the amplitude trajectory: its elevation in ASD reflects the concentrated, repetitive amplitude transitions of a dampened waveform, while its reduction in ADHD reflects a more variable and spatially dispersed amplitude trajectory consistent with the larger b-wave amplitudes reported in that group [2]. Clustering coefficient measures local amplitude-state dwelling; its elevation in ASD indicates prolonged oscillation near specific amplitude levels, consistent with the reduced oscillatory potential amplitudes reported in ASD ERGs [3,7]. Together, these features provide a multi-scale topological fingerprint of the ERG waveform that is directly interpretable in terms of known neurochemical and electrophysiological differences between groups.

Finally, an important conceptual distinction separates the graph-based approach from conventional clustering methods. Traditional clustering algorithms, such as k-means or hierarchical clustering, partition data points into groups based on distance or density in a feature space, treating each observation as an independent vector without encoding relational structure [82]. In contrast, ERG-Graph constructs an explicit relational representation from the raw signal, and features are derived from the resulting topology itself. Spectral clustering bridges these domains by using graph Laplacian eigenvectors for partitioning [83], but its objective remains group assignment. Similarly, community detection on graphs [84] identifies modular structure but still reduces the representation to discrete labels. The ERG-Graph features, by contrast, provide continuous-valued descriptors that characterize signal complexity at multiple topological scales, from local structure (CC, cliques) through mesoscale organization (centrality measures) to global properties (diameter, algebraic connectivity) [48,60,72]. This multi-scale characterization is what enables the ERG-Graph features to capture discriminative information that neither clustering-based reductions nor conventional signal features can access. This approach will support applications to not only retinal responses but also to datasets including cortical responses [55,85] and combined retinal and psychophysical datasets [86] and contributes to the expansion of statistical methods for ERG waveform analysis [87,88,89], thereby supporting the development of objective and non-invasive screening tools for neurodevelopmental disorders.

## 5. Conclusions

This study establishes graph signal processing as a viable and high-performing methodology for extracting discriminative physiological biomarkers from the electroretinogram. By transforming ERG waveforms into topological graph representations, the proposed ERG-Graph framework captures nonlinear signal dynamics that are inaccessible to conventional amplitude-based and spectral methods, yielding substantial improvements in the classification of neurodevelopmental disorders across all evaluated scenarios. The nine graph-theoretic features demonstrated strong statistical separation between diagnostic groups, with large effect sizes, and the most discriminative features carry direct physiological interpretations grounded in known retinal neurochemistry. The framework is computationally lightweight, requiring under 40 ms per waveform, making it suitable for clinical deployment. These results validate the hypothesis that the topology of the ERG amplitude trajectory encodes disorder-relevant information, and provide a principled graph-based foundation for future work integrating deep learning, multimodal biosignals, and prospective clinical validation toward objective, non-invasive screening tools for neurodevelopmental disorders.

## Figures and Tables

**Table 1:** Mean (±SD) graph features by group (Q = 50, 446 Td.s, right eye). Cliff’s δ reported for ASD vs. Control, ADHD vs. Control, and ASD vs. ADHD. Effect size: S = small, M = medium, L = large. *** *p* < 0.001, ** *p* < 0.01.

Feature	Control	ASD	ADHD	*p*-Value	δ ASD/Ctrl	δ ADHD/Ctrl	δ ASD/ADHD
TLC	245.3 ± 42.1	198.7 ± 38.5	278.4 ± 51.2	<0.001 ***	−0.52(L)	0.34(M)	−0.61(L)
THC	312.8 ± 55.6	289.4 ± 49.3	341.2 ± 62.7	0.002 **	−0.22(S)	0.27(S)	−0.41(M)
GNC	18.4 ± 4.2	15.1 ± 3.8	21.7 ± 5.1	<0.001 ***	−0.41(M)	0.36(M)	−0.58(L)
Diam.	8.2 ± 1.9	6.8 ± 1.5	9.5 ± 2.3	<0.001 ***	−0.44(L)	0.39(M)	−0.56(L)
Radius	4.5 ± 1.1	3.9 ± 0.9	5.2 ± 1.4	0.001 **	−0.31(M)	0.28(M)	−0.47(L)
CC	0.42 ± 0.08	0.48 ± 0.09	0.37 ± 0.07	<0.001 ***	0.38(M)	−0.33(M)	0.59(L)
APL	3.8 ± 0.7	3.2 ± 0.6	4.3 ± 0.9	<0.001 ***	−0.47(L)	0.31(M)	−0.57(L)
λ_2_	0.35 ± 0.08	0.41 ± 0.09	0.29 ± 0.07	0.003 **	0.35(M)	−0.40(M)	0.55(L)
Density	0.28 ± 0.05	0.32 ± 0.06	0.24 ± 0.05	<0.001 ***	0.37(M)	−0.42(M)	0.54(L)

**Table 2:** Best two-group classification results using ERG-Graph features.

Comparison	Sex	Model	Flash/Eye	BA	F1	Feat.
ASD vs. Ctrl	Male	XGB	446 Td.s/Right	0.91	0.90	7
ASD vs. Ctrl	Female	RF	446 Td.s/Right	0.84	0.83	8
ASD vs. Ctrl	All	XGB	446 Td.s/R + L	0.84	0.83	12
ADHD vs. Ctrl	Male	SVM	446 Td.s/Right	0.83	0.82	6
ADHD vs. Ctrl	Female	RF	446 Td.s/Right	0.88	0.87	7
ADHD vs. Ctrl	All	RF	446 Td.s/R + L	0.83	0.82	11

**Table 3:** Three-group classification (ASD vs. ADHD vs. Control) comparing feature sets. Results obtained using 446 Td.s flash strength with combined right and left eye features, combined sex. TD = time-domain, DWT = discrete wavelet transform, VFCDM = variable-frequency complex demodulation.

Feature Set	Model	BA	F1	Feat.
TD only	AdaB	0.62	0.60	8
TD + DWT	GradB	0.67	0.65	24
TD + VFCDM	XGB	0.70	0.68	32
TD + DWT+VFCDM	XGB	0.70	0.69	45
ERG-Graph only	XGB	0.78	0.76	18
ERG-Graph + TD	XGB	0.81	0.79	22
Full fusion	XGB	0.79	0.77	38

**Table 4:** Four-group classification (ASD vs. ADHD vs. ASD + ADHD vs. Control). 446 Td.s, combined right and left eye, combined sex.

Feature Set	Model	BA	F1	#Feat.
TD + DWT + VFCDM [12]	XGB	0.53	0.51	45
ERG-Graph only	XGB	0.64	0.62	18
ERG-Graph + TD	XGB	0.67	0.65	22
Full fusion	XGB	0.65	0.63	38

## Data Availability

Data available upon reasonable request from the corresponding author.
